# Non‐cytopathic bovine viral diarrhoea virus 2 induces autophagy to enhance its replication

**DOI:** 10.1002/vms3.1052

**Published:** 2022-12-19

**Authors:** Seung‐Uk Shin, Du‐Gyeong Han, Hyung‐Chul Cho, Eun‐Mi Kim, Kyoung‐Seong Choi

**Affiliations:** ^1^ Department of Animal Science and Biotechnology College of Ecology and Environmental Science, Kyungpook National University Sangju South Korea; ^2^ Korea National Institute of Health Cheongju Chungcheongbuk‐do South Korea

**Keywords:** autophagy, bovine viral diarrhoea virus, persistent infection, viral replication

## Abstract

**Background:**

Bovine viral diarrhoea virus (BVDV) is an important viral pathogen that has an economic impact on the livestock industry worldwide. Autophagy is one of the earliest cell‐autonomous defence mechanisms against microbial invasion, and many types of viruses can induce autophagy by infecting host cells.

**Objectives:**

The aim of this study was to identify the role of autophagy in the pathogenesis of non‐cytopathic (ncp) BVDV2 infection.

**Methods:**

Madin–Darby bovine kidney (MDBK) cells were treated with ncp BVDV2, rapamycin, or 3‐methyladenine (MA) and ncp BVDV2 and then incubated at 37°C for 24 h. Cells were harvested, and the effects of autophagy were determined by transmission electron microscopy (TEM), confocal laser microscopy, western blotting and qRT‐PCR. Apoptotic analysis was also performed using western blotting and flow cytometry.

**Results:**

In ncp BVDV2‐infected MDBK cells, more autophagosomes were observed by TEM, and the number of microtubule‐associated protein 1 light chain 3B (LC3B) with green fluorescent protein puncta was also increased. The ncp BVDV2‐infected cells showed significantly enhanced conversion of LC3‐I to LC3‐II, as well as upregulation of autophagy‐related proteins, including ATG5 and Beclin 1, and substantial degradation of p62/SQSTM1. These results are similar to those induced by rapamycin, an autophagy inducer. E2 protein expression, which is associated with viral replication, increased over time in ncp BVDV2‐infected cells. Inhibition of autophagy by 3‐MA in ncp BVDV2‐infected MDBK cells downregulated the expressions of LC3‐II, ATG5 and Beclin 1 and prevented the degradation of p62/SQSTM1. Moreover, the expressions of phosphorylated Akt and procaspase‐3 were significantly increased in ncp BVDV2‐infected cells. In addition, the mRNA level of protein kinase R (PKR) was significantly reduced in ncp BVDV2‐infected cells.

**Conclusions:**

Our results demonstrate that ncp BVDV2 infection induced autophagy in MDBK cells via anti‐apoptosis and PKR suppression. Therefore, autophagy may play a role in establishing persistent infection caused by ncp BVDV.

## Graphical abstract

Ncp BVDV2 infection induced autophagy in MDBK cells via anti‐apoptosis and PKR suppression. Autophagy may play a role in establishing persistent infection caused by ncp BVDV.

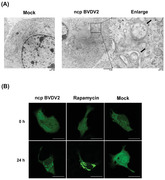

## 1 INTRODUCTION

Bovine viral diarrhoea virus (BVDV) causes significant economic losses worldwide in the cattle industry through decreased productive performance, immunosuppression and persistent infection. BVDV is a single‐stranded positive RNA virus belonging to the genus *Pestivirus*, along with classical swine fever virus (CSFV) and border disease virus in the family Flaviviridae. Based on the 5′‐untranslated region, two BVDV species have been identified: BVDV1 and BVDV2 (Baker, 1995). Each BVDV species is divided into two biotypes, cytopathic (cp) and non‐cytopathic (ncp), based on their ability to cause pathogenic effects in cultured cells (Ridpath et al., 2006). The ncp BVDV is the most prevalent biotype in nature and causes acute and persistent infections. In utero infection of cows during the first 120 days of pregnancy with ncp BVDV strains can result in the birth of persistently infected (PI) calves. These PI animals serve as a major source of viral spread in the herd (Brownlie et al., 1989; Darweesh et al., 2015; Polak & Zmudzinski, 2000). In contrast, cp BVDVs are relatively rare; however, when PI calves are superinfected with a cp BVDV strain, they may develop lethal mucosal disease.

These two viruses interact differently with the host innate immune response. The ncp BVDV evades the host adaptive and innate immune response to establish persistent infection. This may partly be due to the fact that ncp BVDV interferes with the induction of interferon (IFN) α/β (IFN type I) synthesis, the inhibition of which is associated with intracellular viral RNA accumulation (Baigent et al., 2002; Charleston et al., 2001; Gil et al., 2006; Schweizer & Peterhans, 2001; Vassilev & Donis, 2000). The lack of IFN production in ncp BVDV–infected cells might be advantageous for the survival of the virus, as it may prevent the stimulation of innate immune responses (Peterhans & Schweizer, 2014). In contrast, cp BVDV kills infected cells via apoptosis and induces IFN expression, which may be closely related to the execution of apoptosis (Grummer et al., 2002; Schweizer et al., 2006).

Autophagy is a quality‐control system that degrades unwanted cytosolic components, such as damaged organelles and intracellular pathogens, and recycles the degradation products to maintain cellular homeostasis (Jordan & Randall, 2012; Zhou et al., 2017). Autophagy is also involved in viral pathogenesis and plays an important role in innate antiviral response. Interestingly, viruses have evolved to escape or use autophagic pathway for their own benefit; for example, many RNA viruses exploit autophagy for replication. Autophagosome formed during autophagy can provide a physical platform for viral replication machinery (Choi et al., 2018). Recently, complex interactions between autophagy and pathogens have been reported, and viral infections have been shown to induce autophagy, which serves as an innate immune mechanism against viruses, such as Sindbis virus and herpes simplex virus type 1 (Choi et al., 2018). However, the interplay between autophagy and viruses is extremely complex, and the success of many viruses depends on the subversion and sequestration of host autophagic responses (Deretic & Levine, 2009).

Recent studies have reported that ncp BVDV infection induces autophagy and significantly elevates the expression of autophagy‐related genes in Madin–Darby bovine kidney (MDBK) cells (Rajput et al., 2017; Zhou et al., 2017). However, the relationship between autophagy and ncp BVDV infection is not well understood. Therefore, this study aimed to investigate the mechanisms of autophagy induced by ncp BVDV2 and to identify the role of autophagy in the pathogenesis of ncp BVDV2 infection. These results provide useful information for understanding the pathogenesis of ncp BVDV2.

## 2 MATERIALS AND METHODS

### 2.1 Cell culture and virus inoculation

MDBK cells and the virus strain were provided by the Animal and Plant Quarantine Agency (Gimcheon, South Korea) and cells were cultured in MEM‐α supplemented with 10% heat‐inactivated horse serum (Gibco, Waltham, MA, USA), antibiotic/antimycotic (Gibco) and 2 mM L‐glutamine (Gibco) at 37°C under 5% CO_2_. MDBK cells were tested to be negative for BVDV infection prior to being used in this study. The virus strain used in this study was confirmed to be ncp BVDV2a by sequencing analysis (Seong et al., 2013), and this virus was low virulent. The ncp BVDV2a virus strain was obtained after 2 days of culture and centrifuged at 3000 × *g* for 10 min to remove cellular debris. The supernatant was frozen at −80°C until used and virus titration was performed as previously described (Reed & Muench, 1938).

### 2.2 Transmission electron microscopy

MDBK cells (5 × 10^4^/well) were incubated with mock (medium alone) or ncp BVDV2 (100 TCID_50_) at 37°C for 24 h. After washing with PBS, cells were harvested and fixed in 2.5% glutaraldehyde. Then, the cells were washed with PBS twice and post‐fixed in 1% osmium tetroxide at 4°C for 1 h. Next, the cells were dehydrated in a series of ethanol washes followed by being incubated in propylene oxide at 4°C and embedded in epoxy resins. The ultrathin sections were stained with uranyl acetate and lead citrate and then observed using an H‐7100A transmission electron microscopy (TEM; Hitachi, Ibaragi, Japan). Staining and observation of samples were conducted in the Animal and Plant Quarantine Agency (Gimcheon).

### 2.3 Confocal laser microscopy

MDBK cells (5 × 10^4^/ well) were seeded in an eight‐well chamber and incubated at 37°C for 6 h. Then, monolayer MDBK cells (approximately 80% confluent) were transfected with a recombinant plasmid expressing light chain 3 (LC3) fused to green fluorescent protein (GFP‐LC3) using a Lipofectamine 2000 reagent according to the manufacturer's protocol and then incubated at 37°C for 36 h. Cells were then treated with rapamycin (100 nM, Sigma, St. Louis, MO, USA), an autophagy inducer, ncp BVDV2 (100 TCID_50_) or medium alone (negative control) and incubated at 37°C for 24 h. The cells were observed using confocal laser microscopy (LSM700; Carl Zeiss Microscopy GmbH, Germany) and the images were analysed using ImageJ (National Institutes of Health, Bethesda, MD, USA).

Autophagosome formation was detected using an Autophagy Detection Kit (Abcam, Cambridge, UK), according to the manufacturer's instructions. First, MDBK cells (5 × 10^4^) were seeded into an eight‐well chamber and treated with mock infection, rapamycin, ncp BVDV2 or 3‐methyladenine (MA, 1 mM, Sigma), an autophagy inhibitor at 37°C for 24 h. Cells were washed twice with assay buffer and two fluorescent dyes, one for nuclei staining (blue) and the other for autophagy detection (green), were added to each well and then incubated at 37°C for 30 min. Cells were then fixed with 4% formaldehyde and washed three times with the assay buffer. Fluorescent autophagic vacuoles were analysed by confocal microscopy (Carl Zeiss, Jena, Germany). Increased green fluorescence intensity indicates an increase in the degree of autophagy.

### 2.4 Western blotting

MDBK cells (8 × 10^5^/well) were seeded in six‐well plates. Later, ncp BVDV2, rapamycin and 3‐MA were then added to monolayer MDBK cells and incubated at 37°C for 24 h. After washing with PBS, the cells were harvested, resuspended in a lysis buffer (Thermo Fisher Scientific, Waltham, MA, USA) and assayed for protein content using bicinchoninic acid protein assay (Thermo Fisher Scientific). Samples containing equal amounts of protein were separated on 8%, 10%, 12% and 18% sodium dodecyl sulphate–polyacrylamide gel electrophoresis gels and transferred to nitrocellulose membranes then blocked with 5% nonfat dry milk in PBS containing 0.5% Tween 20. After blocking, the membranes were incubated with primary antibodies against LC3B (1:1000; Cell Signaling Technology; Danvers, MA, USA), p62/SQSTM1 (1:1000; Invitrogen; Waltham, MA, USA), ATG5 (1:500; Novus Biological, Littleton, CO, USA), E2 (1:500; Biorbyt, Cambridge, UK), caspase‐3 (1:1000; Abcam), pAkt (Ser473) (1:1000; Cell Signaling Technology), Beclin 1 (1:1000, Cell Signaling) and β‐actin (1:2000; Santa Cruz Biotechnology, Inc., Dallas, TX, USA). The nitrocellulose blots were washed and incubated with horseradish peroxidase‐conjugated secondary specific for anti‐rabbit or anti‐mouse antibodies (Santa Cruz Biotechnology) at room temperature for 1 h. Immunoreactive bands were detected using enhanced chemiluminescence (GE Healthcare, Chicago, IL, USA) and observed using a Gel Documentation System (Invitrogen). Quantification of expression levels was performed using ImageJ. The blots were treated with Restore PLUS Western Blot Stripping Buffer (Thermo Fisher Scientific) and then reprobed for actin.

### 2.5 Annexin V‐FITC staining

For the flow cytometric detection of apoptotic cells, mock (control medium), rapamycin, ncp BVDV2 or 3‐MAtreated ncp BVDV‐infected cells were harvested, washed in binding buffer and then stained with annexin V‐conjugated fluorescein isothiocyanate (FITC) and propidium iodide according to the manufacturer's instructions (BD Biosciences, Franklin Lakes, NJ, USA). Cells stained with annexin V‐FITC and PI were identified and quantitated by FACS Calibur flow cytometry (BD Biosciences, San Diego, CA, USA). Data acquisition and analysis were performed using the CellQuest software (BD). The cells were gated according to forward scatter and side scatter to exclude debris, clumps and dead cells.

### 2.6 Cell viability assay

MDBK cells (1 × 10^4^/well) were seeded in a 96‐well plate and treated with ncp BVDV2, rapamycin or 3‐MA and incubated at 37°C for 24 h. Next, EZ‐CYTOX (Daeillab, Changwon, South Korea) was added to each well according to the manufacturer's instructions and incubated at 37°C for 2 h. After gentle shaking at room temperature for 1 min, the optical density was measured at 620 nm using a microplate reader (TECAN, Männedorf, Switzerland). Cell viability is presented as a percentage (%) relative to the control group (mock group).

### 2.7 Quantitative real‐time RT‐PCR

Total RNA was extracted from mock‐infected, ncp BVDV2, rapamycin or 3‐MA treated ncp BVDV2‐infected cells using RNAiso plus (Takara Bio Inc., Shiga, Japan). First, cDNA was synthesized using the PrimeScript Reagent Kit (Perfect Real Time) (Takara Bio Inc.) on a Thermal Cycler Dice Real Time System III (Takara Bio Inc.) according to the manufacturer's protocol. Briefly, reaction mixtures were prepared in a 10 μl final volume containing 500 ng of total RNA, 0.5 μl of oligo dT primer, 0.5 μl of PrimeScript RT Enzyme Mix, 2 μl of 5× PrimeScript Buffer and RNase‐free water. The RT reaction was conducted in an automated DNA thermal cycler (Takara) at 37°C for 15 min followed by incubation at 85°C for 5 s. Subsequently, qRT‐PCR was carried out using TB Green Premix Ex Taq II (Takara), and the PCR cycling conditions (40 cycles) were as follows: 95°C for 5 min and 60°C for 30 s. Glyceraldehyde‐3‐phosphate dehydrogenase was used as the internal standard control. Each sample was assayed in triplicate. The relative expression levels of the target genes were calculated using the 2^−ΔΔ^ *^Ct^* method. Primers used in this study are listed in Table 1.

**TABLE 1:** Primers used for RT‐PCR in this study

	Primer	Sequences (5′–3′)	References
Autophagy‐related genes	BECN1	AGTTGAGAAAGGCGAGACAC GATGGAATAGGAACCACCAC	Ma et al., 2020
	LC3B	TTATCCGAGAGCAGCATCC AGGCTTGATTAGCATTGAGC	
	GAPDH	CTTCAACAGCGACACTCA CCAGGGACCTTACTCCTT	
Interferon‐induced genes	Mx‐1	ATCTTTCAACACCTGACCGCG GGAGCACGAAGAACTGGATGAT	Yamane et al., 2008
	OAS1	AGCCATCGACATCATCTGCAC CCACCCTTCACAACTTTGGAC	
	PKR	GTTGGGATGGGCATGATTATG AACGTTTGTCTGGCTTCTTGC	
	GAPDH	GATTGTCAGCAATGCCTCCT GGTCATAAGTCCCTCCACGA	Takino et al., 2016

Abbreviations: GAPDH, glyceraldehyde‐3‐phosphate dehydrogenase; LC3B, light chain 3B; OAS1, oligoadenylate synthetase 1; PKR, protein kinase R.

### 2.8 Statistical analysis

Data are expressed as mean ± standard deviation. Each value represents the result of three independent experiments. Statistical analysis was performed using GraphPad Prism version 5.0 for Windows (GraphPad Software Inc., San Diego, CA, USA). Statistical significance was determined using two‐ or one‐way analysis of variance post hoc followed by a least‐squared difference test. *p* ≤ 0.05 was considered significant.

## 3 RESULTS

### 3.1 Ncp BVDV2 infection triggers autophagy in MDBK cells

**FIGURE 1:**
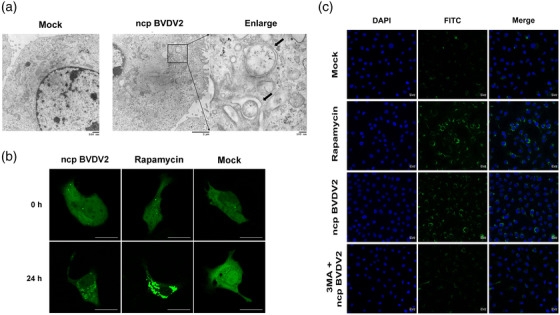
Non‐cytopathic (ncp) BVDV2 infection increases the formation of autophagosome‐like vesicles: (a) Madin–Darby bovine kidney (MDBK) cells were treated with medium alone (negative control) or infected with ncp BVDV2 (100 TCID_50_) and subjected to transmission electron microscopy at 24 h post‐infection to observe double membrane vesicles (black arrow). (b) MDBK cells transfected with green fluorescent protein‐light chain 3 (GFP‐LC3) plasmid for 36 h were treated with ncp BVDV2 (100 TCID_50_), rapamycin (positive control, 100 nM) or medium for 24 h. The number of GFP‐LC3 puncta (green) was analysed by laser confocal microscopy. (c) Representative fluorescence images show the increased presence of autophagic vacuoles in ncp BVDV2‐infected MDBK cells (100 TCID_50_). Cell nuclei were stained with blue. The fluorescence images were captured using laser confocal microscopy. 3‐MA (inhibitor, 1 mM) suppressed autophagic vacuoles in ncp BVDV2‐infected cells. The data are representative of three independent experiments.

To determine whether ncp BVDV2 can induce autophagy in MDBK cells, we infected MDBK cells with ncp BVDV2 and examined autophagosome using TEM. The formation of autophagosome‐like vesicles was observed at 24 h after infection. The presence of double‐membrane vesicles (DMVs) is a hallmark of autophagy induction (Blanchard & Roingeard, 2015; Longatti & Tooze, 2009). Several DMVs were observed in the cytoplasm of ncp BVDV2‐infected MDBK cells, whereas DMVs were not found in mock‐infected cells (negative control) (Figure 1a). DMVs were clearly associated with ncp BVDV2‐mediated autophagy. Moreover, autophagic vacuoles and severe swelling of mitochondria were formed only in ncp BVDV2‐infected MDBK cells compared to mock infection (Figure 1a).

Microtubule‐associated protein 1 LC3 is associated with autophagosome membranes after processing and is essential for the elongation of autophagic vesicles. LC3s have two forms (LC3‐I and LC3‐II), which are generated by post‐translational processing in cells. LC3‐I is cytosolic, whereas LC3‐II is membrane‐bound and is a useful marker of autophagic membranes (Kabeya et al., 2000; Kuma et al., 2007). To investigate whether autophagy was induced in ncp BVDV2‐infected cells, MDBK cells were transfected with GFP‐LC3 and subsequently infected with ncp BVDV2. GFP‐LC3 puncta was observed as ring‐shaped structures using laser scanning confocal microscopy (Figure 1b). Compared with mock infection, the number of GFP‐LC3 puncta increased at 24 h in MDBK cells after infection with ncp BVDV2 or after rapamycin treatment, an autophagy inducer (positive control).

To further determine the effect of autophagy on ncp BVDV2, infected MDBK cells were treated in the presence or absence of 3‐MA, which inhibits autophagy by blocking autophagosome formation. The activation of autophagy in these cells was analysed using an autophagy assay (a fluorescence‐based assay). Rapamycin was used as a positive control. Compared with mock infection, the fluorescent intensity, which represented the autophagic vacuoles, was markedly increased in ncp BVDV2‐infected MDBK cells, similar to that observed in rapamycin‐treated cells (Figure 1c). In ncp BVDV2‐infected MDBK cells treated with 3‐MA, the formation of autophagic vacuoles was markedly decreased compared with that in ncp BVDV2‐infected cells. These results demonstrated that ncp BVDV2 infection induces autophagy in host cells.

### 3.2 Ncp BVDV2 infection increases the levels of autophagy in MDBK cells

**FIGURE 2:**
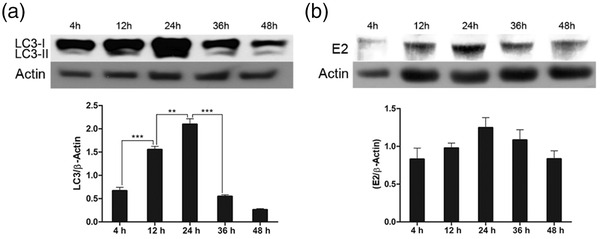
Expression levels of light chain 3 (LC3)‐II and BVDV glycoprotein E2 protein in non‐cytopathic (ncp) BVDV2‐infected cells were determined by western blot analysis. The conversion from LC3‐I to LC3‐II (a) and BVDV E2 (b) were observed in a time‐dependent manner (4, 12, 24, 36 and 48 h). β‐actin expression was used as loading control. The intensity band ratio of each protein to β‐actin was analysed using ImageJ software. The data are representative of three independent experiments.

To investigate whether autophagy was induced by ncp BVDV2 infection, we first evaluated the expression of LC3 in ncp BVDV2‐infected MDBK cells using western blotting. The conversion of LC3‐I to LC3‐II was monitored at 4, 12, 24, 36, and 48 h after ncp BVDV2 infection. As shown in Figure 2a, the conversion of LC3‐I to LC3‐II significantly peaked at 24 h after ncp BVDV2 infection. The expression of E2 protein, which is associated with viral replication, increased over time and peaked at 24 h pi (Figure 2b). The results show that viral replication coincides with ncp BVDV2‐induced autophagy.

**FIGURE 3:**
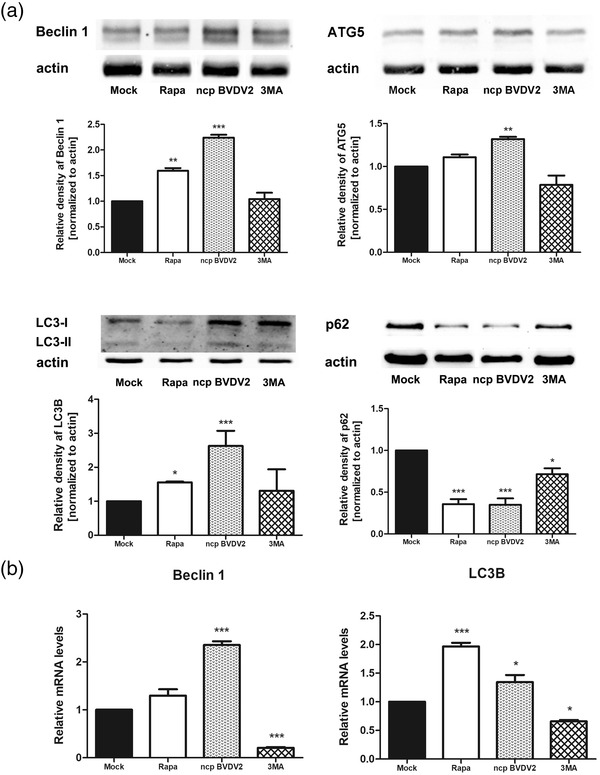
Non‐cytopathic (ncp) BVDV2 infection triggers autophagy in Madin–Darby bovine kidney (MDBK) cells. Autophagy induction was assessed by determining the level of autophagic marker proteins in MDBK cells treated with rapamycin (100 nM), ncp BVDV2 (100 TCID_50_) or 3‐MA (1 mM) for 24 h. (a) Western blot analysis of Beclin 1, ATG5, light chain 3 (LC3)‐II and p62/SQSTM1. β‐actin expression was used as loading control. The intensity band ratio of each protein to β‐actin was analysed using ImageJ software. (b) To further detect the autophagy activity, real‐time quantitative RT‐PCR was performed to determine the mRNA expressions of Beclin 1 and LC3B and normalized to glyceraldehyde‐3‐phosphate dehydrogenase (GAPDH). The results are shown as the mean ± standard deviation (SD) of triplicate experiments. Statistical analyses were performed by a one‐way analysis of variance in GraphPad Prism 5.0 software; *p < 0.05, **p < 0.01 and ***p < 0.001 as compared with the mock‐infected cells. The data are representative of three independent experiments.

Next, we examined autophagic activity in MDBK cells using rapamycin and 3‐MA. As shown in Figure 3a, Beclin 1 and ATG5 proteins were significantly enhanced in ncp BVDV2‐infected cells compared to mock infection, whereas their expressions were decreased in 3‐MA‐treated ncp BVDV2‐infected cells. In addition, when ncp BVDV2‐infected cells were treated with 3‐MA, minimal conversion of LC3‐I to LC3‐II was observed, and their expression levels were not significant, unlike in the ncp BVDV2‐infected cells (Figure 3a). The degradation of p62/SQSTM1 is recognized as an indicator of autophagic flux, and so we examined the expressions of p62/SQSTM1 by western blotting after ncp BVDV2 infection. As shown in Figure 3a, ncp BVDV2‐infected MDBK cells showed a significant degradation of p62/SQSTM1 to a similar extent as with rapamycin treatment, and p62/SQSTM1 degradation was substantially decreased in 3‐MA‐treated ncp BVDV2‐infected cells. The mRNA expression levels of Beclin 1 and LC3 by qRT‐PCR were significantly upregulated in ncp BVDV2‐infected MDBK cells and were significantly downregulated in 3‐MA‐treated ncp BVDV2‐infected cells (Figure 3b).

### 3.3 Ncp BVDV2 infection does not affect cell viability

The effects of autophagy regulators on cell viability were tested using the EZ‐CYTOX assay. No significant differences in cell viability were observed after treatment with ncp BVDV2, rapamycin or 3‐MA (*p* > 0.05), indicating that these treatments did not affect cell viability.

### 3.4 Autophagy induction reduces apoptosis

**FIGURE 4:**
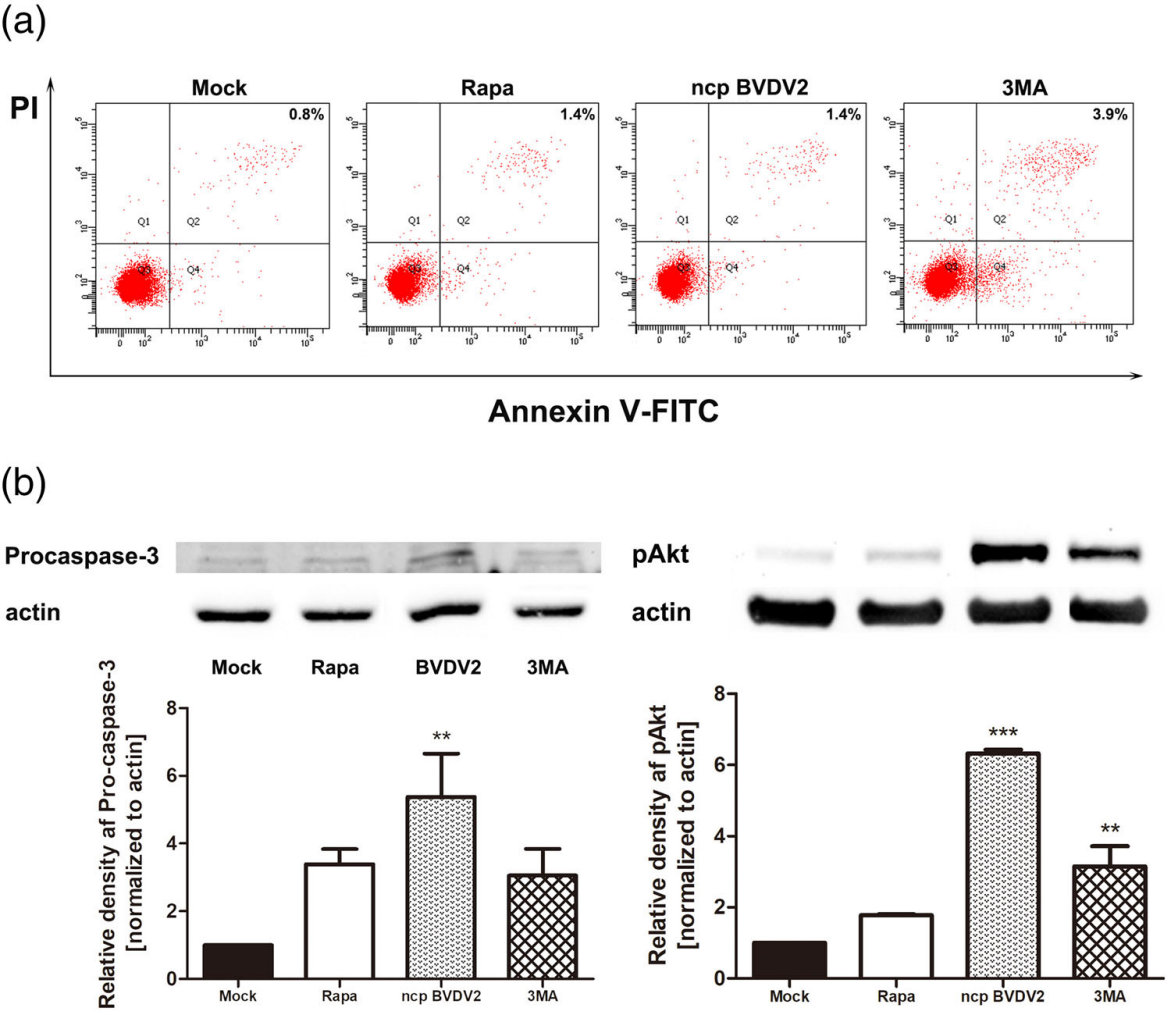
Autophagy regulates apoptosis in non‐cytopathic (ncp) BVDV2‐infected Madin–Darby bovine kidney (MDBK) cells. The cells were treated with rapamycin (100 nM), ncp BVDV2 (100 TCID_50_) or 3‐MA (1 mM) for 24 h. (a) Annexin‐V apoptosis assay. Cells were harvested, stained with annexin V‐fluorescein isothiocyanate (FITC) and propidium iodide and then examined by flow cytometry. (b) Harvested cells were lysed and subjected to western blot analysis using procaspase‐3 and pAkt. β‐actin was used as loading control. The intensity band ratio of each protein to β‐actin was analysed using ImageJ software. The results are shown as the mean ± standard deviation (SD) of triplicate experiments. Statistical analyses were performed by a one‐way analysis of variance in GraphPad Prism 5.0 software; *p < 0.05, **p < 0.01 and ***p < 0.001 as compared with the mock‐infected cells. The data are representative of three independent experiments.

To further define the relationship between autophagy and apoptosis, MDBK cells were treated with rapamycin, ncp BVDV2 or 3‐MA. At 24 h pi, cells were harvested and processed for annexin V‐FITC staining and western blotting. Annexin V staining showed decreased apoptosis in ncp BVDV2‐infected cells, which was similar to mock‐infected cells, whereas ncp BVDV2‐infected MDBK cells treated with 3‐MA showed an increased number of apoptotic cells (Figure 4a).

Caspase‐3 is synthesized as inactive procaspase‐3 (32 kDa) and is cleaved during activation into a small subunit of active caspase‐3 (19 kDa). Activation of caspase‐3 can be determined indirectly by immunoblotting to detect the level of proteolytic cleavage of procaspase‐3. Expression of proteolytically inactive procaspase‐3 (32 kDa) was significantly increased in ncp BVDV2‐infected cells compared to ncp BVDV2‐infected MDBK cells treated with 3‐MA (Figure 4b).

Several signalling pathways (e.g. p38 MAPK and class III phosphatidylinositol‐3 kinase (PI3K/Akt) may induce or inhibit apoptosis (Assefa et al., 2000; Tsuruta et al., 2002), and the activation of these pathways has been previously investigated in BVDV‐infected cells (Bendfeldt et al., 2007). Akt is a well‐characterized anti‐apoptotic signal that promotes survival in many cell types; accordingly, we assessed the phosphorylation of Akt by immunoblotting. Significant phosphorylation of Akt was observed in ncp BVDV2‐infected cells compared to mock‐infected cells (Figure 4b), whereas the expression of pAkt was significantly lower in ncp BVDV2‐infected MDBK cells with 3‐MA treatment. This result shows that Akt signalling may be linked to the inhibition of apoptosis.

### 3.5 Ncp BVDV2 infection downregulated type I IFN‐related genes

**FIGURE 5:**
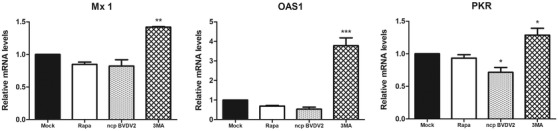
Non‐cytopathic (ncp) BVDV2 infection reduces the expression of type I IFN‐mediated genes. Madin–Darby bovine kidney (MDBK) cells were treated with rapamycin (100 nM), ncp BVDV2 (100 TCID_50_) or 3‐MA (1 mM) for 24 h. The mRNA expressions of *Mx 1*, oligoadenylate synthetase 1 (*OAS1*) and protein kinase R (*PKR*) were determined by quantitative real‐time RT‐PCR and normalized to glyceraldehyde‐3‐phosphate dehydrogenase (GAPDH). *PKR* expression was significantly downregulated in ncp BVDV2‐infected cells. The results are shown as the mean ± standard deviation (SD) of triplicate experiments. Statistical analyses were performed by a one‐way analysis of variance in GraphPad Prism 5.0 software; *p < 0.05, **p < 0.01 and ***p < 0.001 as compared with the mock‐infected cells. The data are representative of three independent experiments.

Ncp BVDVs are known to inhibit IFN‐mediated responses, and the lack of IFNα/β induction facilitates the evasion of the host immune response, leading to cell survival. Expressions of several interferon‐stimulated genes (ISGs), such as myxovirus resistant 1 (*Mx 1*), protein kinase R (*PKR*) and 2′5′‐oligoadenylate synthetase 1 (*OAS1*), are widely used as sensitive markers of antiviral effects (Li et al., 2015; Sadler & Williams, 2008; von Wussow et al., 1990). To determine whether IFN induction was associated with cell survival in ncp BVDV2‐infected cells, we examined the expression of IFN‐inducible factors using real‐time RT‐PCR analysis. The results clearly show that among the three genes examined, the mRNA expression of *PKR* was significantly downregulated in ncp BVDV2‐infected cells compared to mock infection. The expression levels of these genes were significantly upregulated in 3‐MA‐treated ncp BVDV2‐infected MDBK cells (Figure 5).

## 4 DISCUSSION

Autophagy is a cellular physiological mechanism that eliminates and degrades superfluous for damaged organelles as well as invading microorganisms (Chaabane et al., 2013; Mizushima, 2007). It is an essential mechanism for sensing viral infections and antiviral effectors (Jordan & Randall, 2012). Moreover, the survival of viruses is closely related to their ability to counteract autophagy‐associated antiviral defences (Shoji‐Kawata & Levine, 2009). The Flaviviridae family of viruses, such as dengue virus, hepatitis C virus, CSFV and West Nile virus, induces autophagy in infected cells, which is associated with enhanced replication and survival (Beatman et al., 2012; Dreux & Chisari, 2010; Lee et al., 2008; Pei et al., 2016; Shiode et al., 2020). Many studies have shown that BVDV infection can induce autophagy (Fu et al., 2015; Fu, Shi, Ren, et al., 2014; Fu, Shi, Shi, et al., 2014; Rajput et al., 2017; Suda et al., 2019; Fu, Shi, Zhang, et al., 2014; Zhou et al., 2017). However, the specific role of autophagy in ncp BVDV2 infection has not been described. In the present study, our results demonstrate that ncp BVDV2 infection induces autophagy, resulting in enhanced virus replication, inhibition of apoptosis and production of IFN‐mediated antiviral genes. Therefore, this study provides useful information that autophagy plays an important role in the pathogenesis of ncp BVDV2 infection.

Autophagy in mammalian cells is divided into six principal steps: initiation, nucleation, elongation, closure, maturation and degradation (Kang et al., 2011). In general, autophagosome, a double‐membrane structure, is formed after the initiation of autophagy and engulfs cargo such as damaged organelles (Parzych & Klionsky, 2014). Subsequently, the autophagosome combines with the lysosome to form the autolysosome and digests its contents with lysosomal enzymes (Shen & Mizushima, 2014). Our results revealed that ncp BVDV2 infection promoted the formation of DMVs, which are typically associated with the autophagy process. This observation was further supported by the accumulation of GFP‐LC3 puncta and the increased expression of autophagic marker proteins. The autophagic structure induced by ncp BVDV2 infection here differs from previous reports of cp BVDV1 infection, in which cytoplasmic vacuolization is associated with the occurrence of necrosis and necroptosis in host cells (Birk et al., 2008). This may at least partially be explained by the difference in the pathogenesis between the two BVDV genotypes. These results suggest that autophagy can be triggered by ncp BVDV2 infection.

Next, we investigated the expression of key regulators of autophagy, Beclin 1, ATG5 and LC3, during ncp BVDV2 infection. Beclin 1, a critical component of the PI3K complex, is involved in the initial step of autophagosome formation and recruits other autophagy proteins to initiate the formation of the pre‐autophagosomal membrane. ATG5 is indispensable for autophagic vesicle formation, and its knockdown can result in reduction or total inhibition of autophagy, suggesting that it plays a central role in autophagy (Ye et al., 2018). During autophagy, cytosolic protein LC3‐I is combined with phosphatidylethanolamine to become LC3‐II, which is associated with autophagosomal membranes (Kabeya et al., 2000; Kuma et al., 2007). We found that ncp BVDV2 infection significantly enhanced the expressions of Beclin 1, ATG5 and LC3‐II proteins. The increased expression of Beclin 1 and LC3‐II is consistent with the results of previous studies (Fu, Shi, Ren, et al., 2014; Fu, Shi, Shi, et al., 2014). In the present study, the expression of ATG5 was observed in the ncp BVDV2‐infected cells for the first time. ATG5, activated by ncp BVDV2, is believed to facilitate autophagosome membrane maturation. The levels of these autophagy‐related proteins were reduced after 3‐MA treatment of ncp BVDV2‐infected cells; however, these changes were not significant. We further investigated another widely used autophagy marker, p62/SQSTM1, which binds directly to LC3 during autophagy and facilitates degradation of ubiquitinated protein aggregates (Jiang & Mizushima, 2015; Niklaus et al., 2017). Accumulation of p62/SQSTM1 indicates that autophagic flux is blocked (Klionsky et al., 2016). The results show the significant degradation of p62/SQSTM1 in ncp BVDV2‐infected cells. This could be caused by the end of the degradation process of autophagy. Taken together, our findings provide clear evidence that ncp BVDV2 infection induces autophagy in the host cells.

Autophagy plays a pivotal role in the replication of several viruses. The propagation of some viruses is suppressed by autophagy pathways, whereas other viruses exploit autophagy pathways to aid their replication (Ahmad et al., 2018). In this study, the expression of the E2 protein in ncp BVDV2‐infected MDBK cells peaked at 24 h pi. Although we only observed up to 24 h pi, GFP‐LC3 puncta, which are associated with autophagy flux, were increased and autophagosome‐associated LC3‐II protein accumulated after ncp BVDV2 infection. The number of autophagosome was reduced by 3‐MA treatment in ncp BVDV2‐infected cells. This could suggest that autophagosome formation induced by ncp BVDV2 infection may be involved in viral replication and maturation. Similar results have been observed in CSFV infection, where autophagy can be utilized for viral replication and release (Pei et al., 2016). Our findings reveal that ncp BVDV2 utilizes the autophagy process to form DMVs to facilitate virus replication, implying that ncp BVDV2 replication is required for the induction of autophagy. Additional studies are needed to determine the relationship between autophagy and viral propagation.

The interactions among the components of autophagy and apoptosis during viral infection indicate complex crosstalk (Chiramel & Best, 2018; Kudchodkar & Levine, 2009; Nikoletopoulou et al., 2013). Apoptosis is an important mechanism of host defence against viral infections (Everett & McFadden, 1999). It has been shown that, in contrast to ncp BVDV inhibiting apoptosis, cp BVDV induces apoptosis in vitro as well as in vivo (Baigent et al., 2002; Grummer et al., 2002; Schweizer et al., 2006; Zhang et al., 1996). It has been demonstrated that the PI3K/Akt pathway performs a critical function in anti‐apoptosis and autophagy (Jiang et al., 2017; Tsuruta et al., 2002; Wang et al., 2022; Yamaguchi and Wang, 2001). Upon activation through phosphorylation, pAkt promotes cell survival by inactivating the pro‐apoptotic proteins Bad, c‐Raf and caspase‐9 (Burgering & Bos, 1995; Cardone et al., 1998; Franke & Cantley, 1997; Zimmermann & Moelling, 1999) and activates mammalian target of rapamycin (mTOR) phosphorylation (Heinonen et al., 2008). mTOR, a serine/threonine kinase, is a critical downstream target of Akt and plays an important role in the regulation of apoptosis (Hay & Sonenberg, 2004; Sussman et al., 2011). In this study, we found that ncp BVDV2 infection reduced apoptosis, whereas treatment with 3‐MA, inhibitor of PI3K, during ncp BVDV2 infection, triggered apoptosis, as shown by an increase in the surface expression of phosphatidylserine, activation of caspase‐3 and downregulation of pAkt. Although our study did not examine the role of mTOR in ncp BVDV2 infection, the possibility that the PI3K/Akt/mTOR pathway may be involved in anti‐apoptosis mechanisms cannot be excluded. Based on the findings of the crosstalk between autophagy and apoptosis, an autophagic mechanism is likely involved in the inhibition of apoptosis in ncp BVDV2‐infected cells. Therefore, our results indicate that ncp BVDV2 infection can trigger Akt phosphorylation and promote Beclin 1 and LC3‐II to form the autophagosome. Further studies are necessary to identify the interplay between apoptosis and autophagy induced by ncp BVDV2.

Type I IFN is produced by cells in response to viral infection and induces an antiviral state through the regulation of protein synthesis and induction of ISGs (Sadler & Williams, 2008). Several studies have reported that cp BVDV induces IFN synthesis in host cells, whereas ncp BVDV does not, suggesting that this could be a defence mechanism to evade host innate immunity that might be critical for establishing persistent infection (Baigent et al., 2002; Charleston et al., 2001; Gil et al., 2006; Palomares et al., 2013). To date, the mechanism of inhibition of type I IFNs during ncp BVDV infection has not yet been completely resolved. Moreover, the role of type I IFNs in the persistent infection of BVDV remains unclear. During viral infection, a greater production of IFN may play an important role in virus clearance via the activation of innate immunity (Schmeisser et al., 2013). CSFV belongs to the Flaviviridae family and is also able to establish persistent infection. CSFV replication suppresses type I IFN‐inducible antiviral activity and apoptosis by interfering with IFN production, thereby resulting in a persistent survival of the virus in host cells (Bensaude et al., 2004). In the present study, among the IFN‐mediated genes, such as *Mx1*, *OAS1* and *PKR*, which have well‐characterized antiviral activities, ncp BVDV2 infection significantly reduced the expression of *PKR*. This was associated with the inhibition of apoptosis. PKR has been reported to modulate a variety of cellular events, including apoptosis, antiviral state and cell growth rate (Gil et al., 2006). Suppression of PKR activation is critical for efficient replication of many viruses, delaying apoptosis and facilitating the establishment of persistent infection (Katze et al., 2002; Leib et al., 2000). 3‐MA treatment in ncp BVDV2‐infected cells induced the increased expression of *Mx1*, *OAS1* and *PKR*. These effects were related to the reduction in autophagosome and induction of apoptosis. Consequently, our findings suggest that persistent infection caused by ncp BVDV may be closely associated with the suppression of PKR to evade the host immune response, resulting in enhanced viral replication via ncp BVDV2‐induced autophagy. Our results provide important evidence for establishing persistent infection.

In conclusion, the present study demonstrates that autophagy induced by ncp BVDV2 infection plays an essential role in viral replication and inhibits apoptosis and PKR activation. Consequently, autophagy may potentially play a role in establishing persistent infection caused by ncp BVDV. These findings expand our understanding of the pathogenesis of persistent BVDV infection and provide new insights into its control and prevention.

## AUTHOR CONTRIBUTIONS

*Conceptualization*: Kyoung‐Seong Choi. *Methodology*: Seung‐Uk Shin, Du‐Gyeong Han, Hyung‐Chul Cho and Eun‐Mi Kim. *Writing‐original draft preparation*: Seung‐Uk Shin, Du‐Gyeong Han, and Kyoung‐Seong Choi. *Supervision*: Kyoung‐Seong Choi.

## CONFLICT OF INTEREST

The authors report no conflict of interest.

### PEER REVIEW

The peer review history for this article is available at https://publons.com/publon/10.1002/vms3.1052.

## Data Availability

All data generated or analysed during this study are included in the article. Raw data are available upon reasonable request to the corresponding author.
